# Effectiveness of Walking Prescription Using Mobile Health Technology on the Changes in Daily Steps in Older Adults With Cognitive Impairment: Randomized Controlled Study

**DOI:** 10.2196/63081

**Published:** 2025-06-11

**Authors:** Hee Jung Kim, Yun Jung Hwang, Jee Eun Park, Dong Young Lee

**Affiliations:** 1 Biomedical Research Institute, Seoul National University Hospital, Seoul, Republic of Korea; 2 Department of Occupational Therapy, Yeoju University, Yeoju, Republic of Korea; 3 Department of Psychiatry, Seoul National University College of Medicine, 101, Daehak-ro, Jongno-gu, Seoul, 03080, Republic of Korea, 82 2-2072-3155, 82 2-744-7241; 4 Department of Neuropsychiatry, Seoul National University Hospital, Seoul, Republic of Korea; 5 Medical Research Center, Institute of Human Behavioral Medicine, Seoul National University, Seoul, Republic of Korea; 6 Interdisiplinary Program in Cognitive Science, Seoul National University, Seoul, Republic of Korea

**Keywords:** dementia, mild cognitive impairment, mobile health, physical activity, exercise

## Abstract

**Background:**

Walking is frequently recommended as a beneficial physical activity for older adults, as it can enhance both their physical and mental well-being and help prevent cognitive decline and dementia. While it is known that mobile health (mHealth) technology can help improve physical activity among older adults, there is limited research on its effectiveness for older individuals with cognitive impairment.

**Objective:**

This study aimed to determine the effectiveness and feasibility of walking prescriptions using mHealth technology for older adults with cognitive impairment.

**Methods:**

In total, 60 older adults (mean=76.1, SD 5.4) years; female, n=34) with mild cognitive impairment or mild dementia (n=28 and n=32, respectively; Mini-Mental State Examination [MMSE], mean=20.7, SD 4.0) who visited the memory clinic were enrolled. They were randomly assigned into three groups: (1) group A (n=20) was prescribed with a goal of daily steps based on their telemonitored activity using a smart band; (2) group B (n=19) only wore a smart band without a prescription; and (3) group C (n=21) took a monthly education to encourage their walking. All participants took monthly face-to-face sessions with a coach to check their performance and modify the goal of daily steps. Changes in daily steps (primary outcome), cognitive function, physical status, and depressive symptoms from baseline to post-intervention (12 weeks) and follow-up (24 weeks) were assessed by unblinded researchers. Linear mixed effect models with factors of group (reference: control), time (reference: baseline), and their interaction were used for data analysis. Post hoc analyses using paired *t* tests were also conducted.

**Results:**

For group A, there was a significant group × time interaction effect on daily steps both at 12 and 24 weeks (β (SE)=2205.88 (672.34), *P*=.001; β (SE)=2194.63 (884.33), *P*=.015). Group B showed increased numbers of steps only at 12 weeks but not at 24 weeks. Group C showed a continuous decrease in daily steps during the study period. Regarding secondary outcomes, group C showed a significant decline in cognitive function measured by MMSE both at 12 and 24 weeks. However, groups A and B showed stationary MMSE scores during 24 weeks. The number of withdrawn participants did not differ among the 3 groups.

**Conclusions:**

Our findings suggest that walking prescriptions using mHealth technology can effectively increase daily steps in older adults with cognitive impairment.

## Introduction

Physical activity (PA) is protective against age-related cognitive decline and incidence of dementia in older adults [1,2]. It can reduce symptoms of depression and anxiety. It can also increase the psychological well-being of the aging population [3]. Even in older adults diagnosed with mild cognitive disorder (MCI) or dementia, moderate-to-vigorous intensity PA can effectively delay cognitive deterioration and prevent comorbid physical illnesses [4,5]. Therefore, clinical experts have advised older people to exercise regularly and continuously.

Unfortunately, the number of people doing exercise decreases as they get older due to their declined physical ability, lack of knowledge of age-appropriate exercise, false belief that exercise can cause injury, and psychosocial prejudice that older adults should behave carefully and calmly [6]. According to a national survey in Korea, only 37.6% of the population aged 65 years or older maintain PA at a recommended level of above moderate intensity for 150 minutes or more per week, and 46.3% do not exercise at all [7]. A US national survey has also found that only about 27% of individuals aged 65 years or older exercise for the recommended level for physical health [8]. According to a review paper summarizing studies from the United States, Europe, and Japan, 67% of older adults aged 60 years or older spend more than 8.5 hours sitting down [9].

It has been revealed that above moderate-intensity PA can protect or improve cognitive function, even in older adults with neurodegenerative brain diseases [10-13]. A recent study has analyzed the relationship between PA level, cognitive function, and brain pathology of older people using clinical data for 2 years before death and pathology data from brain autopsy and found that exercise and motor ability are associated with higher cognitive function independent of brain pathology, including Alzheimer’s disease [14]. PA can have a counter-effect on degenerative brain diseases by improving brain plasticity and cognitive reserve. However, studies examining the effectiveness of PA programs for older adults with cognitive impairment have been mainly conducted on patients with advanced dementia in long-term care facilities due to limitations of clinic- or community-based programs. It is difficult to monitor PAs of older adults with cognitive impairment and lead them to plan and practice exercise continuously [11,13].

Recent advances in mobile health (mHealth) technology can be used to help individuals form exercise habits on their own or through expert assistance. Continuous monitoring and feedback of PA are possible because mHealth technology allows accurate activity measurements [15] and accumulation of activity information [16-18]. However, many studies on PA programs using mHealth technology have mainly been conducted on young people [19-22]. Although it has also been suggested that PA can be increased in the older population using mHealth technology [23], most studies have been conducted on older adults with physical diseases such as cardiovascular disease, diabetes, and obesity without cognitive dysfunction [23-25]. To the best of our knowledge, few studies have examined the effectiveness of PA programs using mHealth technology in older people with cognitive impairment.

Walking is the most commonly recommended PA. It is also suitable for older adults because it is easy to practice in daily life with few side effects. A relationship between maintaining recommended daily steps in older adults and low mortality has been reported [26]. While most previous studies have reported that moderate to vigorous PA is associated with improved cognitive function [10-13], a cohort study of 78,430 adults found that a higher number of steps (even if less than 10,000 steps per day) was associated with a lower risk of incident dementia [27]. Walking is not only directly influenced by physical functions such as aerobic capacity, balance ability, and limb strength but also by cognitive functions such as attention and executive function [28]. Regular walking can reflect an active lifestyle and self-directed health care. Mild cognitive impairment has been reported to be accompanied by a decrease in gait speed [29] and the decreased interest and motivation often seen in older adults with dementia leads to a decrease in PA [30]. Increasing steps in older adults with cognitive impairment may be a way to compensate for physical and mental frailty.

Clinical practice with prescribing and giving feedback to older patients to exercise at their physical level can lead them to set appropriate goals and practice PA [11,31-34]. While walking education alone has been reported to help increase step counts in older adults [32], it can be more helpful to directly prescribe a target step count based on assessing physical function for older patients with cognitive impairment. In addition, older patients with MCI or dementia may have difficulty remembering or reporting their daily steps; therefore, using mHealth technology to monitor daily step counts is useful to monitor their daily activity. However, simply monitoring daily activity can be insufficient to enhance daily steps; it requires setting appropriate goals, developing strategies to achieve them, evaluating outcomes, and refining the approach, which demands substantial cognitive resources. Consequently, human-led coaching, or personalized guidance, is essential for older adults with cognitive impairment [24]. In this study, mHealth technology is expected to function as a tool for clinicians to modify the daily activity of older patients with MCI or dementia. In other words, clinicians can improve their step counts more effectively if they use mHealth-based monitored data to make personalized prescriptions of steps.

Therefore, this study targeted older adults with cognitive impairment who visited a memory clinic to examine the effectiveness and feasibility of a program for changing daily steps by individualized walking prescription and monitoring using mHealth technology. A 3-arm design was used to compare the use of mHealth technology alone with the addition of human monitoring and feedback.

## Methods

### Participants

In total, 60 participants with mild cognitive impairment or mild dementia (clinical dementia rating [CDR] ≤1) were recruited from a memory clinic at a university hospital. A geriatric psychiatrist confirmed participants’ cognitive diagnosis and evaluated their clinical status via CDR. Diagnoses of MCI and dementia were based on the comprehensive clinical assessments. MCI was defined as individuals who met the core clinical criteria of MCI according to the recommendations of the National Institute on Aging and Alzheimer’s Association guidelines [35]: (1) memory complaint corroborated by self, an informant, or clinician; (2) objective memory impairment for age, education, and gender; (3) largely intact functional activities; and (4) not demented. All MCI individuals had a global CDR score of 0.5. Participants diagnosed with mild dementia met the criteria for dementia in the Diagnostic and Statistical Manual of Mental Disorders 4th Edition (DSM-IV-TR [36]) and CDR score of 0.5 or 1.

Older patients performing no or low level of moderate PA (eg, fast walking for 30 min less than 3 times a week) were included, while those who had at least one of the following were excluded: (1) impossible to walk independently (cane users were not excluded); (2) diagnosed with an above-moderate degree of dementia (CDR ≥2); (3) having a physical illness that might affect their safety during the study period; (4) not being able to use a smart band by oneself or not having caregivers who could help them use it; and (5) participating in other exercise programs within the past year.

The sample size was based on a previous study reporting the effectiveness of the intervention using mHealth technology combined with SMS text messages to increase PA [20]. This study had a similar idea to ours, comparing 3 groups (mHealth-based monitoring with SMS text message vs mHealth-based monitoring without SMS text message vs control). The sample size was conservatively determined to yield a larger number of subjects after comparing 3 groups one by one. For achieving an 80% power (1−*β*=.8) at the 5% level of significance (*α*=.05) with equal allocation, the sample sizes for intervention and active control are 10 and 10, respectively [37]. Then, we doubled the number of subjects to reflect the possibility of a small effect on steps from the intervention, given that the subjects in the reference study had twice as many baseline steps as our participants (9600 vs 4500 steps/day). The drop-out rate was assumed to be 40% since the participants of this study were older adults with cognitive impairment. Therefore, this resulted in 20 subjects in each arm, for a total of 60 subjects.

### Ethical Considerations

This study was approved by the Institutional Review Board of Seoul National University Hospital (H-1708-118-879), and it was registered in a clinical trial registry (KCT0002610). All participants provided written informed consent. To protect privacy, the data used in this study were anonymized before analysis. Participants did not receive any compensation for their participation.

### Study Design

Participants were randomly assigned into 2 intervention groups (group A, walking prescription and using a smart band; group B, only using a smart band) and a control group (group C) at a 1:1:1 ratio. For the randomization, an independent researcher who was not involved in patient evaluation or intervention created a random number table prior to registration of the subject. This study was not blinded even though 2 different researchers conducted baseline and follow-up assessments, respectively.

### Intervention

Our 12-week protocol of intervention was designed for use in a hospital setting. Physicians, nurses, psychologists, physical or occupational therapists, and any other trained paramedic professionals can use this program. In this study, a clinical psychologist and occupational therapist alternatively checked each participant’s performance and compliance with the program and provided educational sessions. Both conducted study procedures uniformly according to the protocol, which included detailed methods of assessment, consultation, and feedback. Prior to the start of the study, a mock coaching session was conducted to validate the interventionists’ adherence to the protocol and feedback skills with the supervision of a physician (a geriatric psychiatrist). Walking education was provided to all participants, including the control group, during the study period. It was conducted in monthly face-to-face sessions, which involved individual guidance for correct posture, appropriate intensity, and a favorable environment for walking.

**Figure 1.**
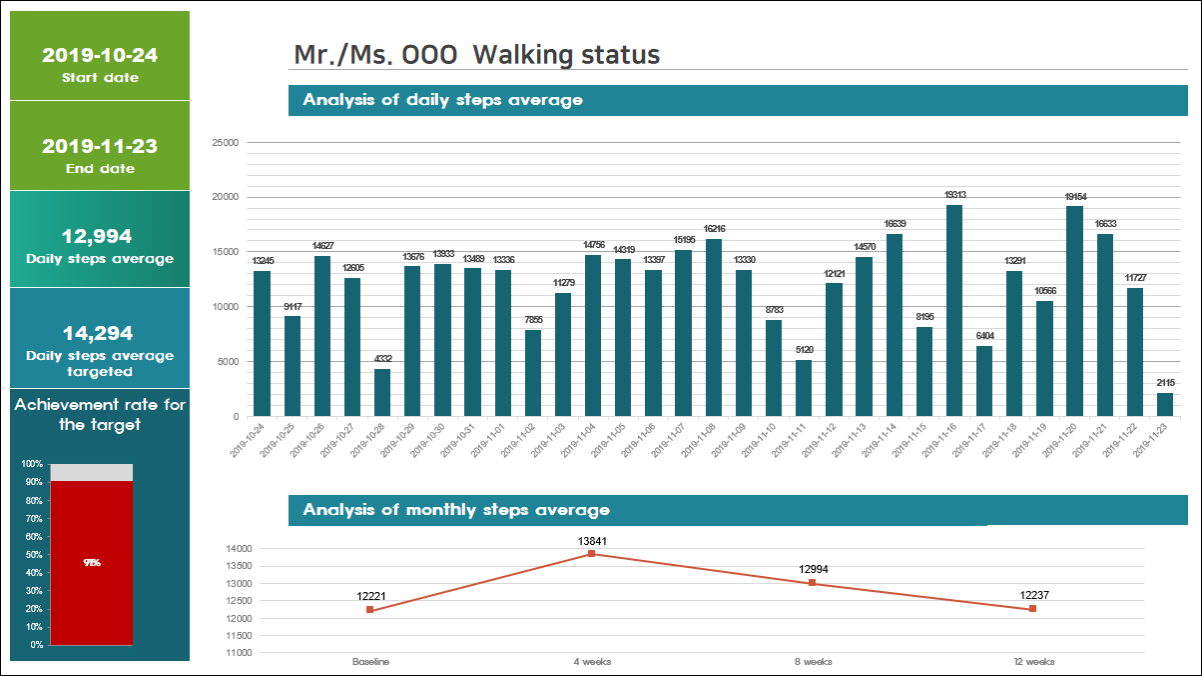
A monthly walking sheet for group A subjects.

Participants in group A wore a smart band and received a personal prescription of daily steps based on their usual walking for the first week. Daily steps were monitored and timely feedback for encouragement was given via telephone or SMS text message at least once a week. If steps were not recorded for 5 consecutive days, the researcher made an emergency call, as this was the deadline to recover data in the event that there was a connection issue between devices. Monthly face-to-face feedback sessions were adjusted depending on individuals’ daily step goals based on their accumulated step data. The target number of steps gradually increased if participants achieved the goal in the previous month, up to the recommended steps by age based on the guidelines for walking in older adults [38]. Participants also discussed with a researcher about their walking tasks, such as when, where, and how much they were walking. A solution was found together in case they had problems in performing tasks. A graph sheet based on telemonitored data was used for these sessions (Figure 1). Even after the 12-week intervention, they were encouraged to wear a smart band and keep walking.

Group B participants were also instructed to wear a smart band. However, they were not given a walking prescription. They were also encouraged to keep wearing the band after the intervention period. The control group (group C) received monthly walking education only and was provided with the same protocol as the intervention groups. A smart band was applied only at baseline, 12 weeks, and 24 weeks for assessment. App screenshots can be found in MultimediaAppendices 1^3^.

Subjects who withdrew consent to participate in this study, failed the baseline assessment, missed more than 2 face-to-face visits, or did not obtain activity data for more than 2 weeks were withdrawn early.

### Outcome Assessment and Measures

At baseline, information on demographics, anthropometrics, and histories of physical and mental illnesses was collected. Primary and secondary outcomes were assessed at baseline, postintervention (12 weeks), and follow-up (24 weeks).

#### Primary Outcome

A change in daily steps was the primary outcome of this study. Smart bands (HR LS405-B6 & HR2.5 Gold edition; Seven Elec Co., LTD) were used to measure subjects’ daily steps. Participants downloaded a mobile application that was linked to the wearable device. They could check their accumulated data on steps, which was transmitted automatically to the database. Average daily steps in a week were calculated at baseline, 12 weeks, and 24 weeks.

#### Secondary Outcomes

Secondary outcomes included three domains: physical function, cognitive function, and depressive symptoms. The Short Physical Performance Battery (SPPB) was used to evaluate physical function [39,40]. Cognitive function was measured using the Mini-Mental State Examination in the Korean version of the CERAD assessment packet (MMSE-KC) [41,42]. The Korean version of the Geriatric Depression Scale [43-45] was used for evaluating depressive symptoms in older patients.

### Statistical Analyses

Analysis of variance (ANOVA) and *χ*^2^ tests were used for continuous and categorical variables, respectively, to compare baseline characteristics among groups. A modified intent-to-treat approach was used to examine effectiveness of the intervention. The analysis included all randomly assigned participants with at least 1 post-baseline observation. Linear mixed-effect models with factors of group (reference: control), time (reference: baseline), and their interaction were used to examine group differences in changes of primary and secondary outcomes from baseline to 12- and 24-week follow-ups. To analyze the primary outcome, age, sex, education, cognitive function, and depressive symptoms at baseline were included in the model as covariates. In addition, we conducted a 2-way mixed ANOVA, where missing data of drop-out subjects were conservatively input as “no change” by carrying forward previous assessment values. For post hoc analyses, time-related changes within each group were examined using paired 2-tailed *t* test (within-group analyses). Whether different patterns were observed by participants’ cognitive status (MCI and mild dementia) was also tested. All statistical analyses were performed with SPSS software (version 23.0; SPSS Inc.).

## Results

**Figure 2.**
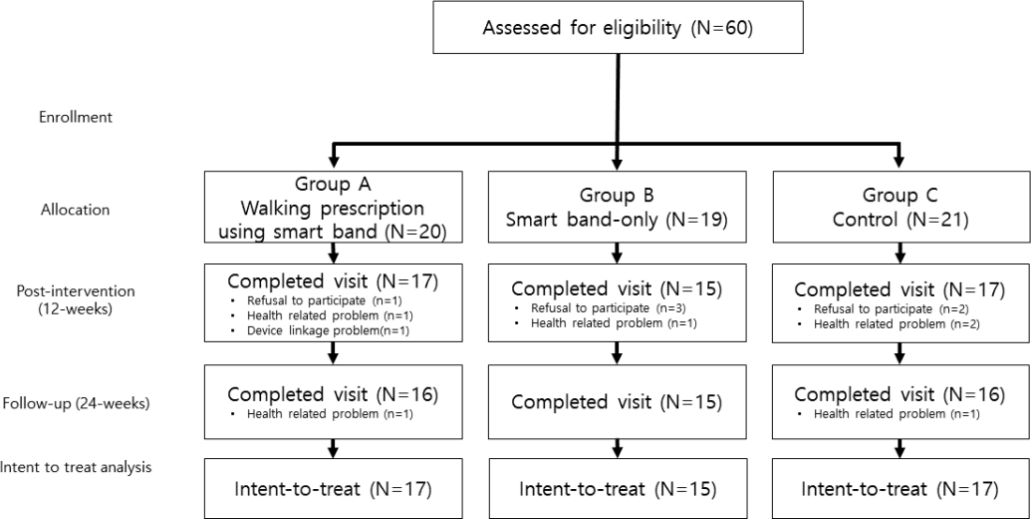
Flowchart for enrollment, allocation, and participation.

In total, 60 older patients diagnosed with MCI or mild dementia were enrolled in this trial and randomly allocated into three groups. Among them, 13 participants dropped out throughout the study period. They did not attend post-baseline assessments. The number of withdrawn participants did not differ among the three groups. Drop-outs were caused by refusals, poor device operation, or health problems. Figure 2 shows the overall study flow, allocation, drop-out, and reasons for withdrawal. As one participant dropped out from group B soon after allocation, a person on the waitlist was allocated to group C with an independent randomization procedure. No adverse events were reported during the study period.

Table 1 shows baseline characteristics of participants according to group allocation. Participants had a mean (SD) age of 76.07 (5.43) years and 11.38 (5.10) years of education. Of them, 34 (57%) were females. Among the three groups, there were no significant differences in demographics or baseline measures such as daily steps, physical status, cognitive function, or depressive symptoms.

A linear mixed-effect model showed a significant group-by-time interaction for the primary outcome measure with adjustment of age, sex, cognitive function, physical status, and depression (Table 2).

**Table 1.** Baseline characteristics of participants.

	Group A (n=20): walking prescription wearing smart band	Group B (n=19): wearing smart band only	Group C (n=21): control	*P* value
Demographics				
Age, years, mean (SD)	74.50 (7)	77.37 (4.15)	76.38 (4.57)	.31
Female, n (%)	12 (60)	11 (58)	11 (52)	.88
Education, years, mean (SD)	10.80 (4.83)	11.95 (5.75)	11.43 (4.95)	.79
Primary outcome				
Daily steps, n (SD)	4439.40 (3443.42)	4122.00 (1743.89)	5157.67 (2305.32)	.3
Secondary outcomes				
Physical status				
SPPB^a^, scores (SD)	8.10 (1.80)	7.63 (1.92)	7.71 (1.95)	.93
Cognitive function				
MMSE-KC^b^, scores (SD)	20.80 (3.44)	19.32(4.93)	21.71 (3.20)	.16
Depressive symptom				
GDS-K^c^, scores (SD)	12.00 (6.00)	12.11 (7.59)	12.19 (5.86)	>.99

a SPPB, Short Physical Performance Battery.

b MMSE-KC, Mini-Mental State Examination in the Korean version of the CERAD assessment packet.

c GDS-K, Korean version of Geriatric Depression Scale.

**Table 2.** Linear mixed-effect models for differences in primary and secondary outcomes.

Intervention group^a^	Primary outcome	Secondary outcomes		
	Daily stepsβ (SE)^b^	Physical status	Cognitive function	Depressive symptom
		SPPB^c^	MMSE-KC^d^β (SE)^b^	GDS-K^e^
Constant	17,067.35 (4741.57)	13.29 (3.90)	3.70 (3.75)	1.53 (5.83)
Walking prescription with a smart band	−817.46 (853.10)	0.24 (0.66)	−0.30 (0.80)	−0.30 (1.35)
Smart band only	−97.67 (897.63)	−0.10 (0.70)	−0.35 (0.84)	0.02 (1.41)
Time^f^				
12 Weeks	−639.94 (475.42)	0.35 (0.29)	−1.76 (0.65)	−0.47 (1.20)
24 Weeks	−1541.50 (625.43)	0.16 (0.40)	−1.43 (0.76)	−2.29 (1.33)
Interaction of group by time				
12 Weeks—Smart band+Prescription	2205.88 (672.34)^g^	−0.12 (0.41)	1.29 (0.92)	0.29 (1.70)
24 Weeks—Smart band+Prescription	2194.63 (884.33)^h^	−0.15 (0.56)	0.88 (1.07)	1.79 (1.88)
12 Weeks—Smart band only	1715.21 (694.39)^h^	−0.02 (0.42)	2.36 (0.95)^h^	−0.06 (1.76)
24 Weeks—Smart band only	1088.30 (905.86)	−0.02 (0.57)	2.83 (1.10)^h^	3.96 (1.93)^h^

a Control as a reference group.

b Linear mixed model, including factors of group, time, and group x time interaction.

c SPPB, Short Physical Performance Battery.

d MMSE-KC, Mini-Mental State Examination in the Korean version of the CERAD assessment packet.

e GDS-K, Korean version of Geriatric Depression Scale.

f Baseline as a reference time.

g *P*<.001.

h *P*<.05.

**Figure 3.**
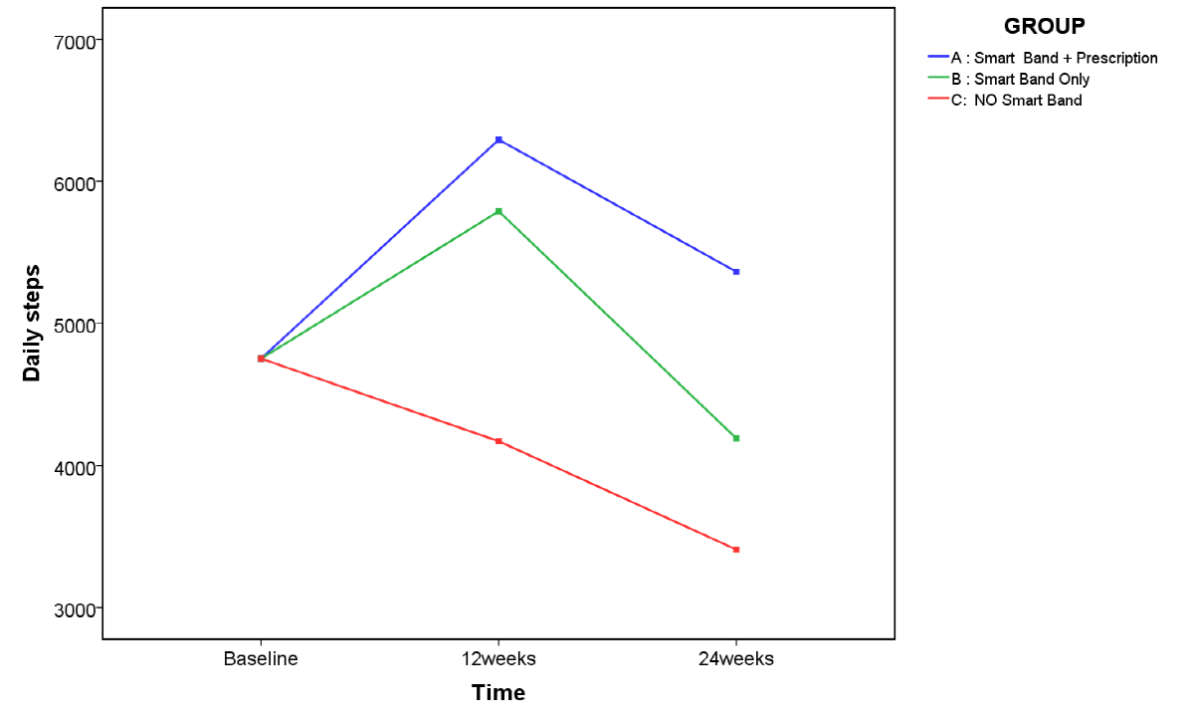
Daily steps by groups using 2-way mixed analysis of variance adjusted with baseline steps. Two-way mixed model analyses of covariance, in which missing data of follow-up were conservatively substituted by the value measured immediately before, included factors of group, time, and group × time interaction, and adjusted for individuals’ daily steps at baseline. Significant group-by-time interaction was shown in daily steps (F(3.38, 2,146,208.77)=3.66, *P*=.01). The raw data on daily steps (mean (SD)) without adjustment of individuals’ daily steps at baseline are as follows: group A: 4553.18 (3598.33) at baseline, 6119.12 (4218.33) at 12 weeks and 5235.76 (3626.98) at 24 weeks; group B: 4449.07 (1708.51) at baseline, 5524.33 (2870.45) at 12 weeks, and 3995.87 (1629.55) at 24 weeks; group C: 5218.12 (2512.09) at baseline, 4578.18 (2425.97) at 12 weeks, and 3707.59 (1799.96) at 24 weeks.

Daily step numbers were significantly increased in group A (walking prescription wearing a smart band) at 12 weeks and 24 weeks, whereas group B (wearing a smart band only) showed an improvement only at 12 weeks, with the increase not sustained till the follow-up. Meanwhile, a continuous decrease in daily steps was observed in group C (control) during the study period. Differences of changes in daily steps by groups were confirmed with a 2-way mixed ANOVA, in which missing data were conservatively substituted with “no change” by carrying forward values. Analyses were adjusted for baseline daily steps (Figure 3; *F*(3.38, 2,146,208.77)=3.66, *P*=.01).

Post hoc within-group analyses repeatedly showed a differential pattern of daily step changes according to intervention (Table 3). Step numbers in group A showed significant increases at 12 and 24 weeks. Although fewer steps were observed at follow-up compared to post-intervention timing, daily steps after 24 weeks were still more than those at the baseline. Participants in group B tended to walk more after wearing a smart band at 12 weeks than at baseline, whereas their daily steps decreased at 24 weeks below their baseline numbers of steps. Finally, group C participants showed a continuous decrease in daily steps over 6 months.

**Table 3.** Intra-group changes in daily steps and cognitive function from baseline to 12 weeks and 24 weeks follow-up.

	Group A(Smart band-based walking prescription, n=16)		Group B(Smart band only, n=15)		Group C(Control group, n=16)	
	Changes at 12 weeksEstimated mean difference(95% CI)^a^	Changes at 24 weeksEstimated mean difference(95% CI)^a^	Changes at 12 weeksEstimated mean difference(95% CI)^a^	Changes at 24 weeksEstimated mean difference(95% CI)^a^	Changes at 12 weeksEstimatedmean difference(95% CI)^a^	Changes at 24 weeksEstimated mean difference(95% CI)^a^
Primary outcome						
Daily steps	1,549,000^a^ (510.279 to 2587.721)	610.438 (−614.599 to 1835.474)	1075.267 (2400.059 to−249.525)	−453.200 (−1502.691 to 596.291)	−788.000 (−2089.440 to 513.444)	−1713.000^a^ (−2761.966 to−664.034)
Secondary outcomes						
MMSE-KC^b^	−0.125 (−1.485 to 1.235)	−0.438 (−2.077 to 1.202)	0.600 (−1.136 to 2.336)	1.400 (−.627 to 3.427)	−1.875^a^ (−2.991 to −.759)	−1.500^a^ (−2.834 to 0.166)
GDS-K^c^	−.500 (−2.170 to 1.710)	−.750 (−2.841 to 1.341)	−.533 (−3.070 to 2.003)	1.667 (−1.123 to 4.457)	−.625 (−4.692 to 3.442)	−2.250 (−4.857 to 0.357)

a Significance at *P*<.008 with Bonferroni correction.

b MMSE-KC: Mini-Mental State Examination in the Korean version of the CERAD assessment packet.

c GDS-K: Geriatric Depression Scale-Korean.

Among secondary outcomes, cognitive function measured by MMSE-KC was changed differently by groups. A significant interaction between groups and time was shown in group B (Table 2). However, this result was not replicated in 2-way ANOVA in which missing data were conservatively substituted with their forward values (*F*(4, 4.04)=1.73, *P*=.15). In post hoc intra-group analyses, a significant decrease in MMSE-KC score was found in the control group at 12 weeks and 24 weeks. On the other hand, cognitive function in groups A and B was maintained at a level similar to the baseline (Table 3).

For depressive symptoms (Korean version of the Geriatric Depression Scale), a significant group-by-time interaction was revealed in group B at the follow-up point, although it was not confirmed in 2-way ANOVA (*F*(4, 11.51)=1.62, *P*=.07). In post hoc within-group analyses, a significant decrease in depressive symptoms was found in MCI individuals of group C at 24 weeks.

## Discussion

### Principal Findings and Comparison With Previous Works

This study examined the effectiveness and feasibility of mHealth-assisted walking prescriptions in older adults with cognitive impairment. It is the first study to demonstrate the effectiveness of a clinic-based PA program using mHealth technology, including people with dementia. Participants who were provided walking prescriptions and feedback based on data from smart bands (group A) showed increased steps in their daily lives both at postintervention (12 week) and follow-up (24 week) periods, whereas participants who were provided smart bands without personal prescriptions (group B) showed an increase in step numbers immediately after the intervention; however, such effect was not sustained at follow-up. Meanwhile, steps of the control group (group C) continued to decrease over the course of this study. As a secondary outcome measure, general cognitive function also showed a decreased pattern in the control group, while participants in groups A and B did not show decreased scores during 6 months, although they were diagnosed with MCI and mild dementia.

Previous studies have reported that walking can improve physical function, such as aerobic capacity [46], and reduce mortality in older adults with cognitive impairment [26]. However, a recent meta-analysis of randomized clinical trials reported that walking did not significantly improve cognitive function in older adults with MCI [46], whereas most previous studies demonstrated that moderate-to-vigorous PA improves cognitive function [10-13]. However, there are other benefits to maintaining adequate step counts for cognitively impaired older adults. Maintaining walking performance, such as pace, has been known to be associated with a lower risk of cognitive decline [47] and improved activities of daily living in older adults [48]. Regular walking can help maintain a robust rest-activity rhythm in cognitively impaired older adults who are vulnerable to circadian disruption, which is associated with the progression of dementia [49]. Since walking itself has cognitive components, including attention and executive function [28], it can stimulate cognitive function in MCI and dementia patients. Maintaining PA is challenging for older adults with cognitive impairment: guiding and monitoring high-intensity PA is nearly impossible in a memory clinic setting. Even though prescribing walking is not sufficient for direct effects on cognitive improvement, it can be a good alternative for maintaining PA for MCI and dementia patients.

A recent meta-analysis has demonstrated that mHealth intervention can increase PA, with such effects maintained for a long term [50]. However, most of the previous studies intervened with young adults. Older adults have many disadvantages when applying mHealth technology. They are less skilled at operating devices. They also have lower levels of digital literacy, making it difficult for them to adopt self-directed exercise programs using mHealth technology. Furthermore, it is almost impossible to have older adults with cognitive impairment, especially those diagnosed with dementia, stick with the exercise on their own. On the other hand, because they regularly visit clinics for treatment, it is easier for clinicians to implement programs in a direct manner. It is important to consider that older adults with cognitive impairment might have difficulty performing complex exercise tasks and accurately recalling their activities. Therefore, this study designed a program that could use mHealth technology to prescribe walking, a simple form of PA, and provide clinician-led monitoring and feedback.

To the best of our knowledge, only a few studies have tested the effectiveness of mHealth-assisted PA intervention in older adults with cognitive impairment. A previous study has confirmed the effects of a mHealth brisk walking intervention in increasing moderate-to-vigorous activity in older people with cognitive frailty [25]. Similar to our study, that study also prescribed and provided feedback on individualized goals. However, it primarily used automated monitoring and feedback generated by a smartphone application. Subjects in that study were recruited from the community. They had mild cognitive impairment with a baseline step count of >12,000 steps. Compared to subjects in our study, who were recruited from hospitals, including those with dementia and those who had a baseline step count of around 4000 steps, subjects in that study were cognitively and physically healthy. Therefore, a relatively self-directed intervention might show effectiveness. Group B subjects of our study were given a smart band and asked to perform self-directed walking. Similar to group A, group B also showed an increase in steps after 12 weeks. However, at the 24-week follow-up, steps tended to decrease back to baseline. That is, the effectiveness of mHealth-guided walking without constant monitoring and feedback was not sustained over a long term in older adults with cognitive impairment. As the previous study only tested the effect after 12 weeks, it was hard to know if the effect was sustained or not. However, the present study’s results suggest that mHealth-supported PA is effective in increasing daily steps when it is accompanied by human-driven feedback, at least in older adults with dementia-level cognitive impairment.

Another previous study has also examined the feasibility and effectiveness of a memory clinic-based walking prescription for individuals with cognitive impairment [24]. In that study, participants did not show an increase in their step count after applying the mHealth-supported walking prescription, similar to our approach. The main difference between that study and ours was the prescribing protocol. The previous study asked participants to double their steps in 6 weeks while our participants were asked to increase their steps by 500 biweekly. If such an increase was not achieved, they repeated the same goal. Our final steps were set at a realistic number based on age-appropriate guideline [38]. In addition, we checked participants’ step counts daily and gave timely feedback at least once a week, whereas the previous study had a fixed biweekly feedback schedule. For older adults with cognitive impairment, the individualized coaching protocol might be more important than the use of mHealth technology itself (such as how to prescribe, monitor, and provide feedback on exercise). Realistic goals, frequent interventions, and flexible approaches might be essential to ensure the effectiveness of the mHealth walking program.

The study also suggests the potential for mHealth-supported walking prescriptions to help maintain cognitive function in older adults with cognitive impairment. Although this program did not improve participants’ cognitive function, MMSE scores of intervention groups (groups A and B) were maintained during the study period, whereas the control group, MCI individuals in particular, showed a significant decrease in this global cognitive function score. A number of previous studies have reported that moderate-to-vigorous PA is an effective modality for maintaining cognitive function in patients with dementia and for improving cognitive plasticity in older adults with MCI [51-53]. Although the mHealth-assisted walking prescription in this study did not increase participants’ cognitive scores, it has the possibility to prevent further deterioration of cognitive function in older adults diagnosed with MCI and early dementia, at least for some time. Given that subjects were already experiencing pathologic cognitive decline, a realistic goal of a clinic-based program might be to delay the rapid progression. At the same time, even though the sample size of this study was calculated to be more conservative than the previous study of similar design, it may have been insufficient to confirm the effectiveness on cognition. Future studies should include a larger number of subjects and different intensities of PA to test the program’s effectiveness on cognitive function.

The mHealth-guided walking prescription in the present study was confirmed to be feasible to use in a clinic-based environment. There were no adverse events in our program. The number of withdrawn participants did not differ among the three study groups. Walking is the most accessible form of PA for older adults. It can be done anywhere and anytime. It is also easy to personalize and monitor individuals’ goals [54]. Techniques we used in this program can be replaced by any application that includes an accelerator. This mHealth-supported walking may be one of the easiest and simplest ways to apply a PA program to older adults having a cognitive problem. Only one dropped out due to difficulty in using a mobile device, although all participants had cognitive impairment.

### Strengths

We designed a 3-arm randomized controlled study. Therefore, we were able to analyze the effectiveness of the program from multiple perspectives: technology use alone versus technology use combined with personalized coaching. We also validated this mHealth-based program in a well-defined population with MCI and mild dementia through comprehensive clinical assessments. Lastly, our program and protocol were confirmed to be feasible in a clinical setting. Only 1 coaching person managed all three study groups’ participants at a time. The coach monitored group A individuals’ steps from the webpage, provided feedback via text or phone at least once a week, and conducted monthly in-person sessions with all participants. Training the coach on the structured protocol to deliver the program took about 4 hours, and the time required for the coach to manage the participants averaged 2-3 hours per day for 6 months. Assuming that there are 20-30 older adults with cognitive impairment over a 6-month period, the program could easily be replicated in other centers with 1 staff member.

### Limitations

This study has several limitations. First, the numbers of participants were small. Although the effectiveness of the primary outcome (changes in step numbers) was confirmed, larger numbers of subjects and differentiated intensity of PA interventions might be needed to confirm the effect on cognitive function. Second, participants and researchers were not blinded to which group individuals were in. However, it was not possible to blind them as different protocols were applied by study groups. In addition, 2 different researchers conducted baseline and follow-up assessments, respectively. Third, the effectiveness of the program could have been different between individuals according to their baseline activity levels. Although we adjusted baseline steps in the analyses of group-level comparison, different effectiveness by individuals needs to be examined in the future. This approach will allow us to develop more personalized guides for PA in older adults regarding the intensity, duration, and interval of exercise. Fourth, the smart band used in this study had yet to be validated. Even though the accelerometer is a simple feature commonly found on smart bands, lack of validation is a limitation of this study. Fifth, apathy may affect motivation and adherence to a PA program. This study assessed depressive symptoms and found no difference in baseline depressive symptoms between participants who completed the program and those who dropped out. However, future research needs to measure apathy, which may directly affect motivation levels, and explore its impact on the effectiveness of the PA program. Lastly, impact of caregivers’ support and assistance on the effectiveness of this program was not assessed. As some caregivers were essential in getting older adults to adhere to our program, it should be included as an important factor in future studies [55].

### Conclusions

Our findings suggest that walking prescriptions using mHealth technology can effectively increase PAs and maintain cognitive health in older adults with cognitive impairment. It is also feasible to apply this mHealth-assisted program to older adults with MCI and mild dementia in the clinic setting. However, the effectiveness of our protocol needs to be confirmed with larger samples and more personalized methods in the future.

## Supplementary material

10.2196/63081 Multimedia Appendix 1 Mobile app screenshot 1: app login page.

10.2196/63081 Multimedia Appendix 2 Mobile app screenshot 2: daily activity including step counts.

10.2196/63081 Multimedia Appendix 3 Mobile app screenshot 3: detailed page for daily steps.

10.2196/63081 Checklist 1 CONSORT-eHEALTH checklist (V 1.6.1).

## Abbreviations

ANOVA: analysis of variance
CDR: clinical dementia rating
DSM-IV-TR: Diagnostic and Statistical Manual of Mental Disorders 4th Edition
MCI: mild cognitive disorder
mHealth: mobile health
MMSE-KC: Mini-Mental State Examination in the Korean version of the CERAD assessment packet
SPPB: Short Physical Performance Battery
