# Supplementary figures and images for: Effectiveness of Walking Prescription Using Mobile Health Technology on the Changes in Daily Steps in Older Adults With Cognitive Impairment: Randomized Controlled Study
Source: JMIR Aging. 2025 Jun 11;8:e63081. doi: 10.2196/63081 (PMC12176243; doi:10.2196/63081)

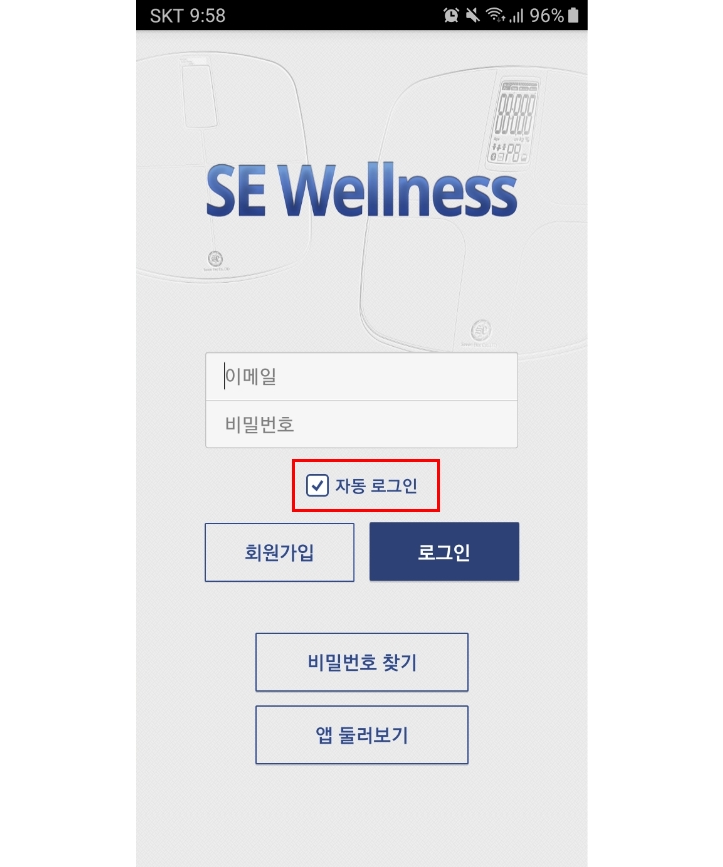

Supplement: Multimedia Appendix 1 [file aging-v8-e63081-s001.png]

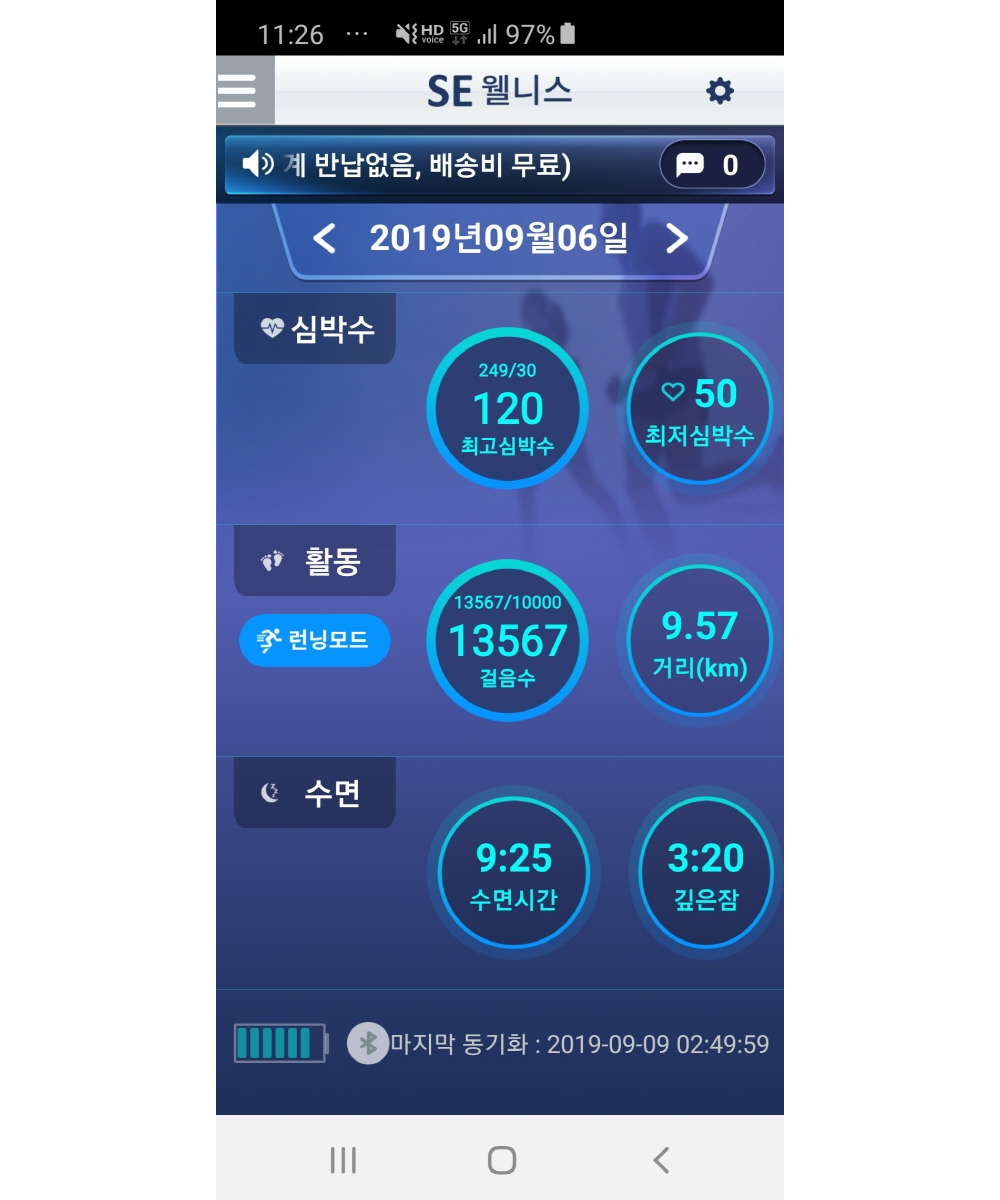

Supplement: Multimedia Appendix 2 [file aging-v8-e63081-s002.png]

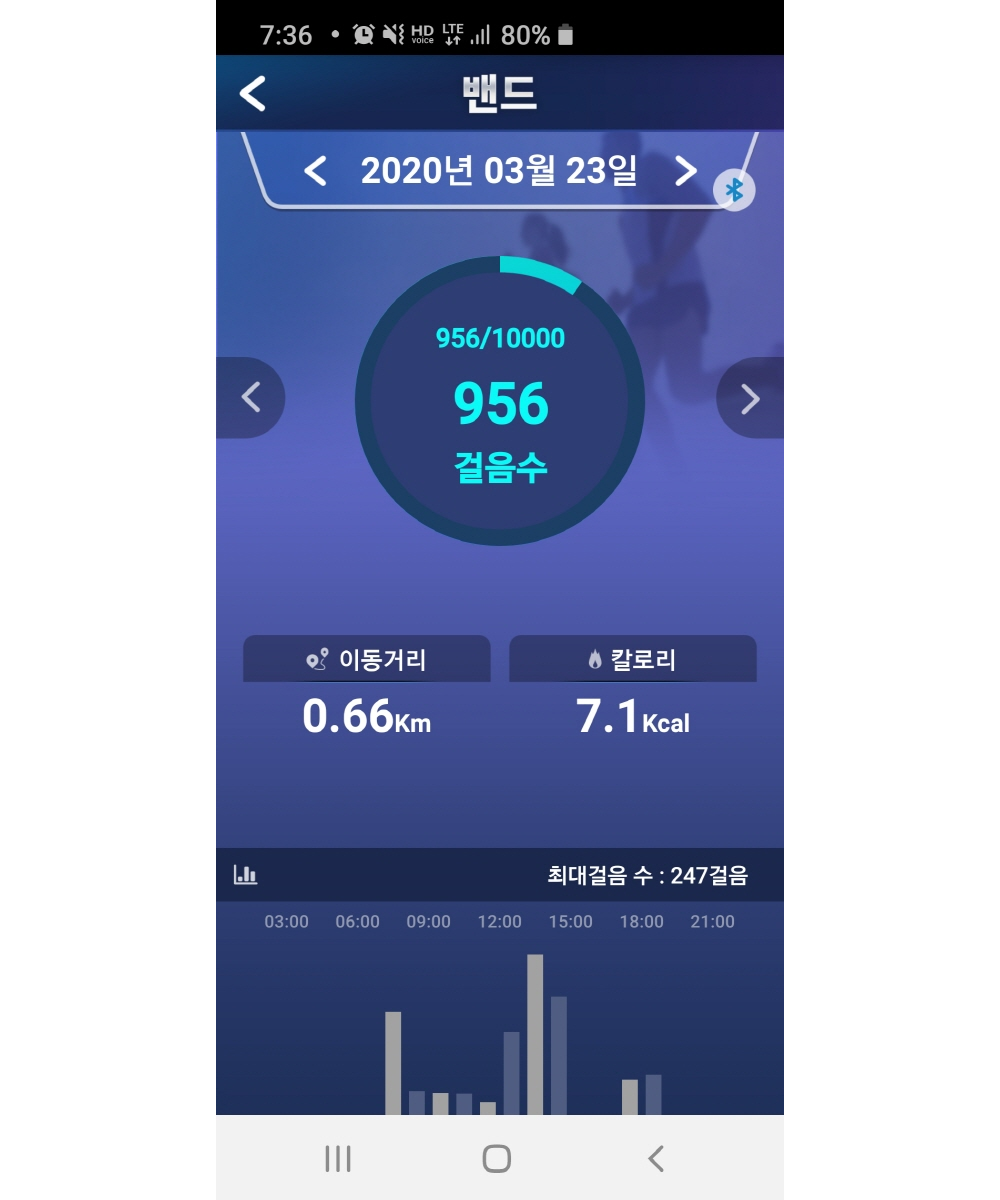

Supplement: Multimedia Appendix 3 [file aging-v8-e63081-s003.png]
